# Effects of elastic tape on kinematic parameters during a functional task in chronic hemiparetic subjects: A randomized sham-controlled crossover trial

**DOI:** 10.1371/journal.pone.0211332

**Published:** 2019-01-25

**Authors:** Gabriela Lopes dos Santos, Erika Shirley Moreira da Silva, Kaat Desloovere, Thiago Luiz Russo

**Affiliations:** 1 Laboratory of Neurological Physiotherapy Research, Department of Physiotherapy, Federal University of São Carlos (UFSCar), São Carlos, SP, Brazil; 2 Instituto de Ciências da Saúde (ICS), Faculdade Alfredo Nasser (UNIFAN), Aparecida de Goiânia, Goiás, Brazil; 3 Department of Rehabilitation Sciences, Faculty of Kinesiology and Rehabilitation Sciences, KU Leuven, Leuven, Belgium; 4 Clinical Motion Analysis Laboratory, CERM, University Hospital Pellenberg, Pellenberg, Belgium; National Center of Medicine and Science in Sport, TUNISIA

## Abstract

**Background:**

Approximately 50 to 70% of post-stroke subjects present a reduction in the upper limb (UL) function even during the chronic phase. An adjuvant technique widely used in neurorehabilitation is elastic taping applications. However, its efficacy in UL treatment for post-stroke subjects still requires further investigation.

**Objective:**

To verify the effects of elastic tape (ET) used on the paretic shoulder in upper limb (UL) performance during a drinking task.

**Method:**

A single-center randomized sham-controlled crossover trial randomized thirteen post-stroke subjects with mild to moderate UL impairment for group allocation to receive first Sham Tape (ST) or first Elastic Tape (ET), with one month of washout. Kinematic measures of a drinking task were taken before and after each intervention (elastic and sham tape), using Three-Dimensional Motion Analysis, and studied using feature analysis and Statistical Parametric Mapping. Outcome measures included spatiotemporal variables, scalar kinematic parameters (starting angles, range of motion—ROM, and endpoint angles) and time-normalized kinematic waveforms of trunk and UL joint angles (scapulothoracic, humerothoracic and elbow).

**Results:**

Elastic tape provided common modifications throughout the task (shoulder more towards midline, reduced scapula protraction and trunk flexion) and important alterations at specific time-instants. At the end of the reaching phase, for both groups (ET and ST), the elastic tape increased elbow extension [ET: CI = 12.57 (6.90 to 18.17), p<0.001; ST: CI: 12.89 (6.79 to 18.98), p<0.001). At the end of transporting the glass to the mouth, patients who underwent the elastic tape intervention presented more shoulder elevation [ET: CI = 16.40 (4.28 to 28.52), p = 0.007; ST: CI: 15.13 (5.79 to 24.48), p = 0.002)]. Moreover, an increase of elbow extension at the end of transporting the glass to the table was observed for both groups [ET: CI = 8.13 (1.48 to 14.79), p = 0.014; ST: CI: 8.20 (4.03 to 12.38), p<0.001)]. However, no changes in the spatiotemporal parameters were observed for both groups during all the phases of the task (p>0.05).

**Conclusion:**

The ET changed UL joint motions and posture during a drinking task in chronic hemiparetic subjects, which defines its role as an adjuvant therapy.

## Introduction

Stroke is the most frequent cause of adult disability worldwide [1]. Approximately 50–70% of stroke survivors in the chronic phase (more than 6 months post-stroke) have upper limb (UL) impairments, such as motor, sensory, perceptual and cognitive deficits. UL impairments may cause limitations in activities of daily living (ADL) and reduce their functional independence, social participation and quality of life [2, 3].

According to the literature, chronic hemiparetic subjects performed slow and non-rectilinear UL movements with a greater number of adjustments along the motion trajectory during functional activities, such as reach-to-grasp and drinking tasks [4–7]. These altered movement patterns are associated with alterations in range of motion (ROM) [5, 7, 8] and in the joint angles at static initial and final positions [4, 6]. These impaired movement patterns could induce disabilities in functional tasks, such as self-care and feeding activities due to difficulty performing reaching, picking up and holding onto objects [2].

Besides these alterations in kinematic scalar parameters, an alternative approach for continuous field analysis, called Statistical Parametric Mapping (SPM), demonstrated that chronic hemiparetic subjects presented a higher number of joint angle alterations at the static starting position and during the reaching phase [6]. Therefore, some common deviations for all phases of the task were observed, including increased scapula protraction, homolateral trunk flexion, and trunk anterior flexion. In addition, these analyses showed reduced elbow extension when a glass was near the table, reduced shoulder elevation in the middle of transporting the glass to the mouth, and a shoulder position that was less toward the midline around the first and last 25% of the time of reaching and returning phases [6].

Given these UL alterations during a functional task and their impact on the movements in stroke survivors, some interventions have been proposed, even in the chronic stroke stage (more than 6 months post-stroke), as some degree of recovery can still be observed in this late-stage phase [9, 10]. An adjuvant technique widely adopted in neurorehabilitation to improve sensorimotor control involves using elastic tape [11–15]. According to the literature, elastic tape applied on the lower limb associated to other types of intervention, such as proprioceptive exercises, potentialize sensory stimulus provided by both intervention strategies, which can influence the motor control [12, 16, 17].

Regarding post-stroke subjects, we recently observed that elastic tape (Kinesio taping) improved the shoulder joint position sense, a submodality of proprioception, with moderate effects compared to non-elastic tape (sham) [11]. Since our previous findings suggested proprioception as an important component of feedforward and feedback control [18], it is assumed that taping may influence motor action. However, studies in the literature related to the effects of elastic tape on UL motor function are controversial [13, 19, 20]. Huang et al. [13] and Kim et al. [19] observed improvements in UL measured by the Fugl-Meyer assessment and the Modified Motor Assessment Scale after elastic taping applications (kinesiology taping), respectively. Instead, Pandian et al. did not observe the effects quantified by Shoulder Pain and Disability Index after elastic adhesive taping applications [20]. The lack of agreement in the literature can be attributed to different methodologies. Furthermore, no studies have verified taping effects on UL performance during functional tasks as improvements in these tasks, such as reaching and drinking, can benefit people’s everyday lives.

Therefore, the aim of the present study was to verify how taping used on paretic shoulder effects the spatiotemporal and joint kinematics parameters during a standardized drinking task in chronic hemiparetic subjects, taking the non-elastic tape condition as a reference. We hypothesized that taping would provide improvements in phase-specific spatiotemporal and joint kinematics.

## Method

### Design

The study was a single-center randomized sham-controlled crossover study. The study was approved by the Ethics Committee in Brazil (Number: 966636) and registered in the Clinical Trials (URL: http://www.clinicaltrials.gov. Unique identifier: NCT02390115). The registration of study was retrospective due to insufficient information for registration, for example, little information regarding the tape application protocol. All participants gave written informed consent.

**Fig 1:**
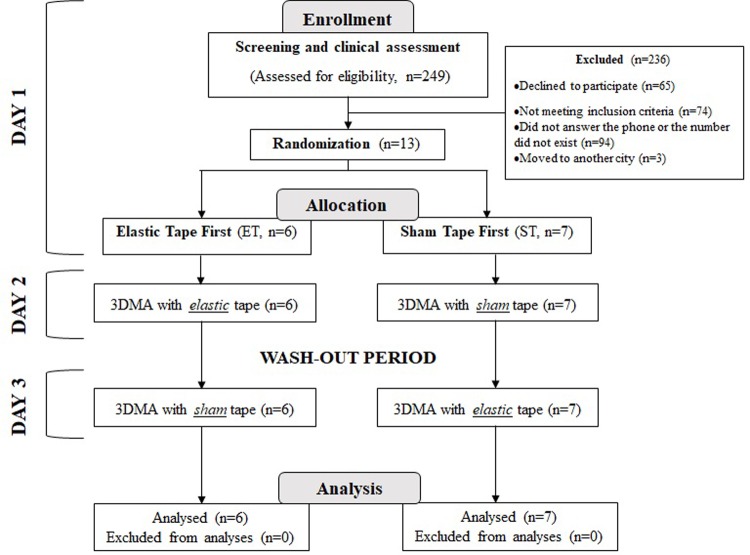
Schematic representation of the experimental design and flowchart.

Assessments were performed at the university and planned over three days. On the first day, a screening and initial evaluation was performed to select the study sample according to the inclusion and exclusion criteria and to characterize the sample. On the same day, an independent staff member randomly assigned participants to one of two groups to receive Sham Tape (ST) first or Elastic tape (ET) first in opaque sealed envelopes. Data analysis was performed by an evaluator who was blinded to the groups. On the other two days, Three-dimensional Motion Analysis (3DMA) (Qualisys Medical AB, Gothenburg, Sweden) of the drinking task was performed without and with the intervention (sham or elastic tape). A wash-out period of one month was given between the second and third evaluation days [11] (Fig 1). All the evaluations were performed in the morning (9 am to 11 am) in a laboratory where the temperature and humidity were controlled.

### Participants

The sample size was calculated using pilot data from four subjects with chronic hemiparesis from each group, using G.Power 3.1 software [21]. For this calculation, we considered the scapula protraction/retraction angle as a primary outcome, because of its involvement in proximal adjustments and its sensitivity to treatment observed in a previous study [6]. The F-test (repeated measures ANOVA, within and between factors) was used and a power of 0.80, alpha of 0.05, and effect size (*η*^2^) of 0.44 were considered. In addition, a loss of 20% of the data was taken into account, requiring a total sample size of 12 participants.

The following inclusion criteria were considered [6, 11]: chronic hemiparetic subjects (post-stroke time exceeding 6 months) due to unilateral ischemic stroke of any hemisphere with lesions restricted to the anterior vascular territory (anterior and medium cerebral arteries) observed in the medical report of the magnetic resonance imaging; aged between 40 and 75 years; minimum score on the Mini Mental State Examination according to the patient’s educational level [22, 23]; proper trunk control, assessed by the ability to remain seated on a chair without trunk and arm support for one minute; spasticity level for shoulder abductor and flexor muscle level of less than 3 on the Modified Ashworth Scale (MAS); and a score of ≥ 30 for the Fugl-Meyer Assessment (FMA-UL) [24]. Moreover, the patients needed to present a minimum passive range of motion of 90° for shoulder flexion, and 30° for shoulder extension and adduction, which were necessary to standardize the use of elastic tape. **A**ll subjects did not participate in any rehabilitation, research and intervention program (such as botulinum toxin) for at least three months.

The exclusion criteria were tape adverse reactions (redness and/or itchiness); skin alterations (ulcers or lesions); diabetes mellitus; understanding aphasia, apraxia, unilateral neglect and/or hemiplegia; botulinum toxin application up to six months before the study; shoulder pain; history of muscle or joint injuries at the shoulder complex or cervical joints (fractures or surgery); any other orthopedic or neurological diseases that affected the data collection; and serious cardiovascular or peripheral vascular disease (heart failure, arrhythmias, angina pectoris or myocardial infarction) [6, 11].

### 3DMA of drinking task

All participants were submitted to 3DMA of a drinking task at the Multidisciplinary Center of Movement Analysis using the optoelectronic ProReflex Motion Capture System (Qualisys Medical AB, Gothenburg, Sweden) with eight high-speed cameras at a sampling frequency of 120 Hz. The analysis was performed by a trained physiotherapist following a previously described standardized protocol [6]. At the beginning of the motion task (starting position), patients were seated with 90° of knee and hip flexion without trunk support and hands pronated on the thigh.

A cluster of markers and eighteen anatomical landmarks were placed bilaterally on the trunk, scapula, upper arm and forearm, following the guidelines of the International Society of Biomechanics (ISB) [25]. Thereafter, static trials were collected to record the reference position and to calibrate the anatomical markers [26]. Ten passive shoulder circumduction movements were performed to determine the glenohumeral joint center [27]. These data collection steps were performed before and after the intervention. Afterwards, the anatomical landmarks were removed, and the participants were instructed to perform a drinking task at a self-selected speed and return to the starting position. The task was repeated three times with the paretic side before and after interventions (six trials per evaluation day) with one-minute rest intervals between trials. One familiarization trial was performed at the beginning of the pre-3DMA.

### 3DMA processing

Data analysis was performed by one evaluator using Qualisys Track Manager Software. The drinking task was divided into four phases: reaching for the glass (including grasping), transporting the glass to the mouth (including sipping), returning it to the table (including releasing the grasp), and returning the hand to the initial position. The onset and end of each phase was visually identified using a frame-by-frame movement inspection [6].

UL kinematics calculations were computed with Upper Limb Evaluation in Motion Analysis (ULEMA) software (https://github.com/u0078867/ulema-ul-analyzer) according to ISB recommendations. Four segments (trunk, scapula, humerus, forearm) were included and eleven joint angles were extracted: trunk (flexion-extension, lateral flexion and rotation), scapula (tilting, rotation, pro-retraction), shoulder (elevation plane, elevation and rotation), elbow (flexion-extension and pro-supination). These parameters were expressed relative to the static posture. Positive values represent flexion, homolateral flexion, and internal rotation for trunk motions; posterior tilting, medial rotation, and protraction for scapula motions; internal rotation; and elbow flexion. Negative values represent opposite motions. For plane of elevation, values near 90° demonstrate motions more in the sagittal plane, and values near 0° represent motions more in the frontal plane.

### Outcome measures

The primary outcome included spatiotemporal and kinematic parameters extracted from the joint angle waveforms, calculated per phase [6]. Spatiotemporal parameters comprised phase duration (PD, second), relative phase duration (%PD, ratio between phase duration and total task duration, expressed in percentage), peak velocity (PV, mm/s), time to peak velocity (%TPV), and trajectory deviation [6] (TD, ratio between the length of the travelled wrist path and the length of a straight line connecting the start and endpoints). The TD variable was not calculated for the return phase because there was no clear target to reach. The extracted kinematic parameters included starting angles, range of motion (ROM, difference between minimum and maximum angle), and joint angles at the point of task achievement (PTA, final angle to complete the task). Secondary outcomes were the time-normalized kinematic waveforms of different angles of trunk, scapulothoracic, humerothoracic and elbow per phase.

### Interventions

A physiotherapist certified in the Kinesio Taping Method made the interventions on the paretic shoulder. For elastic and sham tape intervention, a blue Kinesio Tex Gold Finger Print tape (5 cm wide) and Cremer non-elastic tape (5 cm wide) (Cremer S/A, São Paulo, Brazil) were used respectively. Both interventions were made immediately after the pre-3DMA and were kept during the post-3DMA.

Prior to the application of both interventions, the participants´ skin was cleaned with swabs containing 70% isopropyl alcohol. The application was from the acromioclavicular joint to a point immediately below the insertion of the deltoid muscle to facilitate muscle contraction. Three pieces of the tape were cut, and the application was always initiated by the anterior deltoid followed by the middle and posterior. For all applications, the respective muscles were placed passively in a position of passive stretching. The first tape was placed to the anterior portion of the deltoid with the shoulder at 30° passive extension. The second tape was placed to the middle portion of the deltoid with the shoulder at 30° of passive horizontal adduction. To place the third tape to the posterior deltoid, the limb was positioned at 90° of the passive flexion of the shoulder. The elastic tape was applied with 10–15% of the total elastic tape tension (“paper-off”). The sham tape was placed similarly to the elastic tape with the patient in the same position. After the application, the patient remained seated for 10 minutes [11]. The assessor and the patient were not blinded regarding the intervention due to the color of the tape used, which was a limitation of the study. In addition, it was not possible to cover the limb, as it could have generated sensorial stimuli and impaired the test. However, to mitigate this limitation, an independent evaluator (ESMS) organized the data for statistical analysis.

### Statistical analysis

All extracted kinematic scalars and spatiotemporal parameters showed normal and homogeneous distribution according to the Shapiro-Wilk and Levene tests, respectively. Thus, a two-way ANOVA with repeated measures using Bonferroni’s correction was adopted to verify the effect of interaction (group and evaluation time), main effect of group (ST and ET), and main effect of evaluation time (after and before sham and elastic tape). A significance level was set at 0.05. Moreover, the effect size of interaction was determined through partial eta squared calculation (*η*^2^) [28, 29]. A *η*^2^ around 0.2, 0.5, and 0.8 correspond to a small, medium and large effect, respectively [30]. Effect size of elastic tape intervention was calculated through the mean difference between pre and post- intervention and 95% confidence interval (95% CI) for both groups (ET and ST) [31]. All statistical tests were carried out using SPSS software version 17.0 (SPSS Inc, Chicago, IL, USA).

As complementary analysis, the mean kinematic waveforms of all joint angles for each time-normalized phase were compared before and after the elastic and sham conditions using the Statistical Parametric Mapping (SPM) paired t test, with statistical significance set to alpha at 0.05. In these analyses, the scalar output (SPM{t}) at each normalized time series point was calculated for each SPM t test, which indicates the magnitude of differences. Thereafter, the critical threshold (Tcritical) for which only 5% of the smoothed random curves would be expected to exceed and the probability with which supra-threshold regions could have been produced by a random field process with the same temporal smoothness were calculated [32, 33]. An example of the SPM results is shown in the supplementary material (S1A Fig). SPM analyses were performed using the open-source SPM1d code (version 0.4, http://www.spm1d.org) in MATLAB (R2017b, The Mathworks Inc, Natick, MA).

## Results

### Participants

Patients were recruited from July 2014 to July 2015 from the lists of rehabilitation centers in São Carlos. During this period, 249 post-stroke subjects were assessed for eligibility. However, 65 declined to participate, 74 did not meet the inclusion criteria and 97 were excluded for other reasons, for example, they did not answer the phone or the number did not exist. Thus, 13 subjects were randomly allocated to one of two groups to receive Elastic tape first (n = 6, ET) or Sham Tape first (n = 7, ST). All included patients completed the crossover experiment and the data analysis was successfully conducted for all the participants (Fig 1). Seven patients presented hemiparesis to the right and six to the left without limitations in passive ROM of shoulder (abduction and flexion) and with a spasticity level of 0 to +1 in the shoulder muscles (abductors and flexors) (S1 Table).

### Spatiotemporal and scalar kinematic parameters

There were no interaction effects, main time effect and main group effect for all the spatiotemporal parameters (S1B Table) and for ROM of all joint angles (Tables 1–4) in each phase. On the other hand, the results revealed small to medium interaction effects for starting angles of the elevation plane (p<0.001, *η*^2^ = 0.41, more towards midline), scapula protraction-retraction (p = 0.002, *η*^2^ = 0.33, less protraction), and trunk flexion-extension (p = 0.002, *η*^2^ = 0.37, less flexion) with effects for both groups (Table 1).

**Table 1:** ROM, starting angles, and PTA for all joints assessed while reaching for a glass for both sequences (groups: ET and ST) pre and post-interventions (elastic and sham tape).

		Interventions					
	Sequence	Elastic Tape		Sham Tape		Mean difference (95% CI)	p-value
		Pre	Post	Pre	Post		
***Elevation Plane***							
Start	ET	39.52 (3.29)	47.03 (5.45)*	40.54 (4.64)	39.09 (2.60)	-7.50 (-14.19 to -0.81)	0.025*
	ST	39.74 (1.67)	46.73 (1.18)*	39.16 (3.28)	40.55 (3.49)	-6.98 (-10.52 to -3.44)	<0.001*
ROM	ET	42.34 (6.88)	31.09 (8.54)	41.56 (8.62)	40.74 (15.20)	————	0.351
	ST	41.50 (9.84)	38.21 (11.25)	42.03 (9.34)	41.43 (9.83)	————	1.000
PTA	ET	73.33 (1.70)	84.08 (6.18)*	72.52 (3.68)	74.88 (4.19)	-10.75 (-17.59 to -3.91)	0.002*
	ST	73.22 (3.82)	82.08 (5.65)*	73.35 (2.39)	72.81 (4.24)	-8.86 (-13.76 to -3.95)	0.001*
***Shoulder Elevation***							
Start	ET	-22.80 (2.09)	-21.55 (6.67)	-21.79 (9.84)	-21.72 (9.83)	————	1.000
	ST	-21.56 (8.56)	-21.88 (8.64)	-22.77 (3.04)	-23.48 (5.12)	————	1.000
ROM	ET	34.81 (4.11)	34.02 (3.85)	35.00 (3.74)	36.74 (3.93)	————	1.000
	ST	36.28 (4.54)	37.52 (3.47)	34.97 (4.32)	34.59 (3.67)	————	1.000
PTA	ET	-51.40 (2.58)	-56.25 (12.44)	-52.25 (2.26)	-52.56 (7.85)	————	1.000
	ST	-51.40 (5.04)	-53.57 (7.95)	-51.26 (2.10)	-51.04 (4.16)	————	1.000
***Shoulder Rotation***							
Start	ET	-43.35 (6.29)	-44.35 (12.84)	-44.77 (4.29)	-44.19 (6.09)	————	1.000
	ST	-44.76 (7.31)	-45.15 (7.78)	-43.68 (5.59)	-44.21 (5.37)	————	1.000
ROM	ET	26.17 (5.09)	27.29 (4.90)	26.31 (4.50)	26.12 (4.67)	————	1.000
	ST	26.05 (7.00)	26.26 (5.08)	26.61 (7.53)	27.22 (5.23)	————	1.000
PTA	ET	-56.62 (8.31)	-56.83 (1.60)	-55.11 (5.47)	-56.26 (6.29)	————	1.000
	ST	-56.53 (2.76)	-57.28 (2.35)	-57.35 (4.65)	-56.49 (2.31)	————	1.000
***Scapula Protraction-Retraction***							
Start	ET	34.76 (3.57)	29.30 (2.70)*	34.59 (2.01)	34.24 (2.99)	5.46 (0.41 to 10.51)	0.031*
	ST	35.03 (3.94)	29.80 (2.27)*	34.90 (2.45)	34.31 (3.54)	5.23 (3.02 to 7.45)	<0.001*
ROM	ET	6.70 (2.00)	6.65 (2.08)	6.39 (1.28)	6.41 (1.80)	————	1.000
	ST	6.83 (1.14)	6.45 (1.62)	6.46 (0.81)	6.56 (1.13)	————	1.000
PTA	ET	41.87 (4.51)	36.74 (3.68)*	41.29 (3.39)	41.15 (3.74)	5.13 (0.96 to 9.31)	0.014*
	ST	41.14 (4.20)	36.53 (3.06)*	41.99 (3.82)	41.28 (3.14)	4.61 (1.70 to 7.51)	0.002*
***Scapula Rotation***							
Start	ET	3.81 (2.06)	2.50 (0.80)	3.39 (0.55)	3.18 (0.56)	————	0.404
	ST	3.80 (0.82)	2.72 (0.73)	3.55 (1.06)	3.65 (1.13)	————	0.200
ROM	ET	11.27 (1.42)	11.67 (2.08)	11.32 (0.99)	11.69 (1.64)	————	1.000
	ST	11.72 (2.15)	11.33 (0.82)	11.95 (0.80)	11.51 (1.25)	————	1.000
PTA	ET	-5.03 (0.80)	-7.32 (1.49)*	-5.07 (0.83)	-5.08 (0.68)	2.88 (0.12 to 4.45)	0.036*
	ST	-5.06 (1.30)	-7.08 (0.46)*	-5.06 (1.02)	-5.07 (0.80)	2.02 (0.93 to 3.11)	0.001*
***Scapula Tilting***							
Start	ET	-16.17 (2.97)	-13.08 (2.52)	-16.24 (1.92)	-16.25 (3.17)	————	0.351
	ST	-16.11 (3.92)	-15.68 (3.02)	-16.25 (2.93)	16.09 (3.73)	————	0.200
ROM	ET	7.21 (1.82)	7.87 (2.32)	7.62 (3.11)	7.88 (2.04)	————	1.000
	ST	7.77 (3.26)	7.63 (1.72)	7.42 (2.43)	7.76 (2.84)	————	1.000
PTA	ET	-11.28 (1.32)	-10.09 (0.61)	-11.98 (1.23)	-11.61 (1.87)	————	0.595
	ST	-11.28 (1.16)	-10.57 (2.50)	-11.25 (2.40)	-11.31(2.16)	————	1.000
***Elbow Flexion-Extension***							
Start	ET	71.40 (3.52)	70.44 (19.63)	70.92 (13.16)	70.76 (13.56)	————	1.000
	ST	72.50 (4.14)	71.16 (4.70)	71.96 (8.50)	70.48 (4.76)	————	1.000
ROM	ET	27.28 (7.08)	26.25 (5.56)	26.96 (3.23)	29.85 (7.93)	————	1.000
	ST	27.47 (5.24)	28.31 (3.86)	27.17 (6.90)	27.44 (12.18)	————	1.000
PTA	ET	72.25 (17.10)	59.69 (15.71)*	72.96 (11.37)	75.46 (13.43)	12.57 (6.90 to 18.17)	<0.001*
	ST	72.44 (6.99)	59.55 (6.70)*	73.12 (16.07)	71.17 (6.03)	12.89 (6.79 to 18.98)	<0.001*
***Elbow Pronation-Supination***							
Start	ET	121.39 (5.61)	121.25 (11.42)	122.03 (13.21)	122.14 (14.76)	————	1.000
	ST	122.09 (13.90)	122.20 (12.77)	121.49 (8.16)	121.76 (12.35)	————	1.000
ROM	ET	13.33 (1.29)	13.33 (1.30)	13.98 (1.34)	13.88 (2.75)	————	1.000
	ST	13.29 (1.10)	13.84 (1.11)	13.75 (0.60)	13.35 (1.59)	————	1.000
PTA	ET	112.68 (6.30)	112.84 (7.65)	112.54 (11.10)	112.86 (10.70)	————	1.000
	ST	112.27 (16.33)	113.03 (8.14)	113.96 (3.61)	111.79 (6.97)	————	1.000
***Trunk Flexion-Extension***							
Start	ET	7.27 (0.48)	5.98 (0.81)*	7.41 (1.49)	7.37 (1.10)	1.16 (0.09 to 2.23)	0.031
	ST	7.36 (0.68)	6.20 (0.54)*	7.32 (0.73)	7.57 (0.65)	1.30 (0.29 to 2.31)	0.010
ROM	ET	2.66 (0.45)	2.58 (0.31)	2.65 (1.05)	2.61 (0.76)	————	1.000
	ST	2.71 (0.27)	2.62 (0.81)	2.65 (0.21)	2.61 (0.21)	————	1.000
PTA	ET	7.25 (0.40)	6.20 (0.63)*	7.54 (0.63)	7.43 (0.54)	1.04 (0.50 to 1.57)	<0.001*
	ST	7.33 (0.41)	6.30 (0.62)*	7.18 (0.47)	7.33 (0.69)	1.03 (0.55 to 1.53)	<0.001*
***Trunk Lateral Flexion***							
Start	ET	0.58 (0.23)	0.61 (0.25)	0.63 (0.29)	0.64 (0.23)	————	1.000
	ST	0.62 (0.30)	0.65 (0.21)	0.60 (0.18)	0.62 (0.19)	————	1.000
ROM	ET	2.91 (1.06)	2.91 (0.88)	2.92 (0.72)	2.96 (0.81)	————	1.000
	ST	3.01 (0.60)	2.99 (0.45)	2.89 (0.60)	2.96 (0.65)	————	1.000
PTA	ET	-1.16 (0.65)	-1.01 (0.64)	-1.18 (0.51)	-1.19 (0.80)	————	1.000
	ST	-1.14 (0.89)	-1.04 (0.48)	-1.14 (0.76)	-1.16 (0.62)	————	1.000
***Trunk Axial Rotation***							
Start	ET	0.48 (0.34)	0.46 (0.33)	0.42 (0.26)	0.48 (0.25)	————	1.000
	ST	0.44 (0.23)	0.48 (0.22)	0.44 (0.34)	0.46 (0.33)	————	1.000
ROM	ET	6.89 (1.52)	6.61 (1.03)	6.78 (2.02)	7.24 (2.48)	————	1.000
	ST	6.78 (1.99)	7.21 (1.17)	6.92 (1.48)	6.71 (1.79)	————	1.000
PTA	ET	4.84 (0.47)	5.01 (1.72)	4.86 (2.65)	4.73 (1.42)	————	1.000
	ST	4.84 (2.37)	4.86 (1.69)	4.75 (1.15)	4.79 (1.68)	————	1.000

Data expressed as mean and standard deviation. ET: Elastic tape first. ST: Sham Tape first. Start: Starting angle. PTA: Point of Task Achievement. ROM: Range of Motion. CI: confidence interval (just for variable with significant interaction effect). p-value of comparison between pre and post elastic tape intervention for each group (ET and ST). ————: when p-value of interaction, group and time evaluation effects were not significant.

*p<0.05 compared to pre-ET intervention.

**Table 2:** ROM and PTA for all joints assessed while transporting the glass to the mouth for both sequences (groups: ET and ST) pre and post-interventions (elastic and sham tape).

		Interventions					
	Sequence	Elastic Tape		Sham Tape		Mean difference (95% CI)	p-value
		Pre	Post	Pre	Post		
***Elevation Plane***							
ROM	ET	16.49 (6.77)	14.21 (4.13)	15.55 (6.81)	14.32 (5.83)	————	1.000
	ST	16.26 (4.66)	14.49 (4.40)	15.61 (5.43)	16.16 (3.95)	————	1.000
PTA	ET	60.88 (3.36)	68.51 (2.41)*	62.37 (2.76)	61.25 (4.29)	-7.63 (-11.63 to -3.63)	<0.001
	ST	61.04 (7.39)	69.40 (4.44)*	60.97 (5.21)	60.47 (3.06)	-8.36 (-14.01 to-2.72)	0.004
***Shoulder Elevation***							
ROM	ET	10.47 (2.67)	10.11 (7.57)	10.71 (5.55)	10.83 (2.66)	————	1.000
	ST	10.87 (3.96)	10.75 (6.72)	10.90 (2.89)	10.37 (3.67)	————	1.000
PTA	ET	-44.21 (12.81)	-60.52 (11.49)*	-45.69 (5.47)	-46.21 (4.69)	16.40 (4.28 to 28.52)	0.007
	ST	-44.74 (6.53)	-59.88 (8.30)*	-43.34 (11.92)	-44.42 (9.33)	15.13 (5.79 to 24.48)	0.002
***Shoulder Rotation***							
ROM	ET	14.27 (4.35)	15.39 (4.35)	15.19 (7.88)	14.04 (3.90)	————	1.000
	ST	11.43 (4.02)	14.51 (6.04)	15.18 (5.78)	14.91 (5.22)	————	1.000
PTA	ET	-45.98 (9.15)	-46.30 (12.41)	-45.23 (7.05)	-45.22 (7.37)	————	1.000
	ST	-45.14 (18.03)	-45.22 (20.89)	-46.01 (6.08)	-45.77 (9.77)	————	1.000
***Scapula Protraction-Retraction***							
ROM	ET	3.46 (0.81)	3.11 (1.93)	3.90 (1.50)	3.90 (0.93)	————	1.000
	ST	3.74 (1.51)	3.79 (1.48)	3.62 (1.41)	3.52 (0.68)	————	1.000
PTA	ET	42.27 (2.74)	33.77 (2.39)*	41.59 (3.83)	40.50 (2.94)	-8.50 (-16.64 to -0.36)	0.039
	ST	40.96 (3.01)	35.02 (3.06)*	41.75 (4.99)	41.20 (4.84)	-5.94 (-9.79 to -2.09)	0.003
***Scapula Rotation***							
ROM	ET	5.77 (2.63)	4.16 (1.59)	4.98 (2.02)	4.69 (1.56)	————	1.000
	ST	4.92 (1.76)	5.00 (2.51)	4.89 (1.88)	4.34 (1.42)	————	1.000
PTA	ET	-12.88 (2.24)	-7.89 (2.43)*	-12.73 (2.23)	-13.69 (2.25)	-4.85 (-7.97 to -1.73)	0.002
	ST	-12.85 (3.83)	-8.00 (2.82)*	-12.44 (2.61)	-12.54 (3.13)	-4.99 (-7.90 to -2.08)	0.001
***Scapula Tilting***							
ROM	ET	2.55 (0.71)	2.66 (0.82)	2.94 (0.55)	2.72 (1.29)	————	1.000
	ST	2.30 (0.41)	2.39 (0.59)	2.44 (0.68)	2.86 (0.63)	————	1.000
PTA	ET	-9.66 (4.74)	-9.51 (2.97)	-9.70 (1.45)	-10.70 (2.58)	————	1.000
	ST	-9.73 (4.48)	-9.19 (7.05)	-9.51 (4.16)	-10.14 (3.95)	————	1.000
***Elbow Flexion-Extension***							
ROM	ET	56.08 (13.85)	57.97 (6.68)	59.12 (11.15)	58.43 (8.03)	————	1.000
	ST	54.52 (7.78)	58.18 (9.94)	58.89 (6.11)	59.35 (10.83)	————	1.000
PTA	ET	127.27 (8.45)	126.78 (2.57)	125.96 (9.65)	128.22 (5.96)	————	1.000
	ST	125.96 (9.64)	128.22 (5.96)	127.53 (4.96)	125.30 (6.30)	————	1.000
***Elbow Pronation-Supination***							
ROM	ET	15.56 (2.54)	17.87 (3.23)	17.72 (3.16)	18.27 (2.88)	————	1.000
	ST	16.58 (4.39)	16.88 (4.24)	15.39 (2.50)	16.32 (2.35)	————	1.000
PTA	ET	103.53 (10.95)	104.79 (6.73)	105.06 (11.47)	107.18 (9.29)	————	1.000
	ST	104.96 (15.83)	104.65 (9.18)	102.64 (11.06)	104.48 (9.42)	————	1.000
***Trunk Flexion-Extension***							
ROM	ET	2.18 (0.69)	2.19 (0.96)	2.36 (0.69)	2.44 (1.03)	————	1.000
	ST	2.20 (1.14)	2.14 (0.89)	2.26 (0.85)	2.15 (0.63)	————	1.000
PTA	ET	4.34 (0.97)	2.37 (0.80)*	4.49 (0.96)	4.33 (0.77)	1.98 (0.74 to 3.21)	0.002
	ST	4.28 (0.90)	2.33 (0.75)*	4.28 (0.70)	4.32 (1.00)	1.94 (0.38 to 3.50)	0.013
***Trunk Lateral Flexion***							
ROM	ET	2.56 (1.50)	3.46 (1.15)	2.66 (1.49)	2.44 (1.10)	————	1.000
	ST	2.72 (1.26)	2.24 (0.85)	2.63 (0.79)	2.46 (1.43)	————	1.000
PTA	ET	0.57 (2.99)	0.54 (2.74)	0.53 (2.45)	0.53 (2.49)	————	1.000
	ST	0.45 (3.25)	0.50 (2.66)	0.52 (2.77)	0.62 (2.55)	————	1.000
***Trunk Axial Rotation***							
ROM	ET	6.27 (2.79)	6.25 (1.86)	6.45 (2.37)	6.62 (1.97)	————	1.000
	ST	6.16 (1.08)	6.07 (0.95)	6.40 (1.01)	5.77 (1.21)	————	1.000
PTA	ET	1.80 (1.42)	1.80 (0.90)	1.81 (1.00)	1.80 (0.99)	————	1.000
	ST	1.81 (1.00)	1.80 (0.99)	1.78 (0.75)	1.76 (0.66)	————	1.000

Data expressed as mean and standard deviation. ET: Elastic tape first. ST: Sham Tape first. Start: Starting angle. PTA: Point of Task Achievement. ROM: Range of Motion. CI: confidence interval (just for variable with significant interaction effect). p-value of comparison between pre and post elastic tape intervention for each group (ET and ST). ————: when p-value of interaction, group and time evaluation effects were not significant. NS: not significant (interaction, group and time evaluation effects).

*p<0.05 compared to pre-ET intervention.

**Table 3:** ROM and PTA for all joints assessed while transporting the glass to the table for both sequences (groups: ET and ST) pre and post-interventions (elastic and sham tape).

		Interventions					
	Sequence	Elastic Tape		Sham Tape		Mean difference (95% CI)	p-value
		Pre	Post	Pre	Post		
***Elevation Plane***							
ROM	ET	10.07 (2.56)	10.94 (2.36)	9.85 (2.18)	10.61 (2.94)	————	1.000
	ST	10.81 (4.14)	9.89 (1.00)	10.23 (3.15)	10.95 (2.65)	————	1.000
PTA	ET	71.29 (0.83)	80.31 (1.40)*	72.11 (3.82)	71.06 (2.42)	-9.02 (-12.89 to -5.15)	<0.001
	ST	71.20 (2.76)	79.94 (3.98)*	71.34 (0.60)	71.71 (4.16)	-8.73 (-13.04 to -4.43)	<0.001
***Shoulder Elevation***							
ROM	ET	10.62 (4.07)	10.91 (1.82)	10.34 (2.87)	10.93 (3.89)	————	1.000
	ST	10.63 (1.83)	10.86 (4.85)	10.96 (3.03)	10.68 (1.59)	————	1.000
PTA	ET	-54.87 (4.00)	-54.23 (1.41)	-54.97 (2.31)	-54.68 (3.01)	————	1.000
	ST	-54.47 (3.48)	-54.43 (1.59)	-54.21 (2.92)	-54.83 (2.94)	————	1.000
***Shoulder Rotation***							
ROM	ET	11.58 (3.98)	11.47 (2.13)	11.45 (3.42)	11.12 (4.08)	————	1.000
	ST	11.13 (4.14)	11.36 (3.99)	11.91 (6.38)	11.23 (6.46)	————	1.000
PTA	ET	-61.74 (12.60)	-62.57 (12.13)	-61.63 (9.88)	-62.49 (9.64)	————	1.000
	ST	-62.88 (11.24)	-61.69 (10.30)	-61.17 (11.22)	-61.81 (11.52)	————	1.000
***Scapula Protraction-Retraction***							
ROM	ET	4.32 (1.21)	4.22 (0.55)	4.51 (1.80)	4.36 (1.66)	————	1.000
	ST	4.24 (0.83)	4.16 (1.37)	4.18 (1.04)	4.30 (1.36)	————	1.000
PTA	ET	45.40 (4.78)	37.98 (6.47)*	45.15 (3.42)	44.58 (2.94)	7.43 (3.51 to 11.34)	0.001
	ST	45.22 (2.64)	37.36 (2.97)*	45.73 (2.98)	44.76 (4.19)	7.86 (1.30 to 14.41)	0.016
***Scapula Rotation***							
ROM	ET	4.26 (1.60)	4.21 (0.42)	4.49 (1.47)	4.11 (0.86)	————	1.000
	ST	4.34 (0.78)	4.28 (0.85)	4.12 (1.55)	4.31 (1.38)	————	1.000
PTA	ET	-11.56 (2.95)	-11.51 (2.22)	-11.68 (2.22)	-11.72 (2.56)	————	1.000
	ST	-11.97 (2.24)	-11.48 (3.01)	-10.96 (2.91)	-11.06 (4.63)	————	1.000
***Scapula Tilting***							
ROM	ET	2.73 (0.80)	2.75 (0.72)	2.76 (0.91)	2.73 (1.08)	————	1.000
	ST	2.73 (0.59)	2.72 (0.67)	2.75 (0.84)	2.70 (0.40)	————	1.000
PTA	ET	-11.93 (2.46)	-11.50 (1.41)	-10.86 (1.97)	-11.41 (1.19)	————	1.000
	ST	-11.09 (2.08)	-11.36 (0.72)	-11.44 (3.16)	-11.27 (2.03)	————	1.000
***Elbow Flexion-Extension***							
ROM	ET	58.14 (9.08)	58.79 (9.36)	58.55 (8.60)	58.03 (8.25)	————	1.000
	ST	58.25 (7.89)	58.08 (7.53)	58.49 (8.34)	58.91 (8.56)	————	1.000
PTA	ET	69.43 (3.16)	61.30 (2.40)*	68.61 (3.86)	69.98 (3.86)	8.13 (1.48 to 14.79)	0.014
	ST	69.56 (5.38)	61.36 (3.12)*	69.27 (3.03)	69.16 (7.53)	8.20 (4.03 to 12.38)	<0.001
***Elbow Pronation-Supination***							
ROM	ET	12.76 (4.86)	12.83 (5.13)	12.52 (3.20)	12.95 (5.02)	————	1.000
	ST	12.63 (4.90)	12.38 (3.47)	12.78 (5.42)	12.81 (5.75)	————	1.000
PTA	ET	115.09 (9.93)	116.25 (14.75)	115.40 (12.41)	115.31 (8.56)	————	1.000
	ST	115.99 (9.95)	116.28 (10.56)	115.29 (10.59)	114.74 (14.46)	————	1.000
***Trunk Flexion-Extension***							
ROM	ET	2.99 (1.02)	2.82 (0.73)	3.32 (0.81)	3.34 (1.43)	————	1.000
	ST	2.87 (0.94)	2.87 (1.09)	2.86 (1.14)	2.89 (1.46)	————	1.000
PTA	ET	7.82 (3.39)	4.07 (1.99)*	7.33 (3.02)	8.40 (2.37)	3.74 (1.21 to 6.27)	0.004
	ST	6.25 (1.37)	4.25 (1.19)*	6.23 (2.41)	6.22 (2.07)	2.00 (0.34 to 3.65)	0.016
***Trunk Lateral Flexion***							
ROM	ET	2.08 (0.82)	2.31 (0.59)	1.95 (1.50)	1.85 (0.80)	————	1.000
	ST	2.06 (1.06)	2.02 (0.63)	1.94 (0.73)	1.82 (1.02)	————	1.000
PTA	ET	1.33 (0.49)	1.15 (0.52)	1.22 (0.24)	1.32 (0.59)	————	1.000
	ST	1.31 (0.58)	1.29 (0.53)	1.24 (0.46)	1.40 (0.58)	————	1.000
***Trunk Axial Rotation***							
ROM	ET	4.45 (0.60)	4.29 (1.07)	4.08 (0.90)	4.37 (3.89)	————	1.000
	ST	4.31 (1.71)	4.32 (1.11)	4.17 (1.47)	3.90 (1.70)	————	1.000
PTA	ET	5.16 (1.02)	4.95 (0.95)	4.91 (1.25)	4.95 (0.83)	————	1.000
	ST	4.81 (1.49)	4.70 (1.60)	5.22 (1.37)	4.96 (1.71)	————	1.000

Data expressed as mean and standard deviation. ET: Elastic tape first. ST: Sham Tape first. Start: Starting angle. PTA: Point of Task Achievement. ROM: Range of Motion. CI: confidence interval (just for variable with significant interaction effect). p-value of comparison between pre and post elastic tape intervention for each group (ET and ST). ————: when p-value of interaction, group and time evaluation effects were not significant. NS: not significant (interaction, group and time evaluation effects).

*p<0.05 compared to pre-ET intervention.

**Table 4:** ROM and PTA for all joints assessed while returning to the initial position for both sequences (groups: ET and ST) pre and post-interventions (elastic and sham tape).

		Interventions					
	Sequence	Elastic Tape		Sham Tape		Mean difference (95% CI)	p-value
		Pre	Post	Pre	Post		
***Elevation Plane***							
ROM	ET	49.03 (11.68)	47.34 (11.67)	48.45 (10.20)	48.62 (11.22)	————	1.000
	ST	49.32 (12.53)	47.99 (8.73)	48.66 (13.48)	48.30 (12.74)	————	1.000
PTA	ET	19.99 (12.19)	35.72 (17.21)*	21.55 (11.35)	23.49 (11.97)	-15.73 (-30.54 to -0.92)	0.034
	ST	21.96 (12.07)	36.13 (12.50)*	18.11 (11.76)	20.63 (10.73)	-14.18 (-20.15 to -8.20)	<0.001
***Shoulder Elevation***							
ROM	ET	35.30 (4.08)	35.98 (2.37)	35.17 (5.33)	36.08 (1.93)	————	1.000
	ST	35.81 (6.69)	33.52 (2.42)	35.17 (7.26)	35.72 (5.34)	————	1.000
PTA	ET	-18.91 (2.81)	-20.66 (3.30)	-22.35 (5.26)	-18.53 (3.23)	————	1.000
	ST	-19.12 (3.86)	-16.79 (1.73)	-18.85 (2.09)	-21.62 (1.94)	————	1.000
***Shoulder Rotation***							
ROM	ET	28.79 (3.13)	28.30 (0.92)	28.20 (9.32)	29.46 (4.92)	————	1.000
	ST	26.61 (2.66)	26.95 (4.42)	26.79 (7.38)	29.36 (5.54)	————	1.000
PTA	ET	-35.44 (11.52)	-35.23 (6.10)	-37.34 (5.73)	-36.36 (6.04)	————	1.000
	ST	-36.68 (6.10)	-35.32 (4.84)	-35.82 (5.06)	-36.30 (4.81)	————	1.000
***Scapula Protraction-Retraction***							
ROM	ET	6.96 (2.09)	6.55 (2.02)	7.38 (1.91)	7.55 (2.23)	————	
	ST	6.82 (1.63)	7.37 (2.08)	6.37 (1.81)	7.05 (1.85)	————	
PTA	ET	38.14 (1.93)	32.61 (2.60)*	38.40 (5.53)	37.60 (5.85)	5.53 (1.15 to 9.91)	0.012
	ST	39.57 (3.88)	30.86 (1.34)*	38.11 (6.35)	38.16 (5.81)	8.70 (0.47 to 16.94)	0.036
***Scapula Rotation***							
ROM	ET	11.87 (1.60)	12.70 (1.18)	12.66 (4.26)	10.93 (1.28)	————	1.000
	ST	12.01 (3.87)	12.14 (2.02)	11.80 (2.03)	12.23 (2.25)	————	1.000
PTA	ET	-1.65 (2.22)	-1.81 (1.58)	-1.77 (3.18)	-1.55 (2.99)	————	1.000
	ST	-1.51 (4.65)	-1.29 (4.83)	-1.61 (3.83)	-1.39 (3.53)	————	1.000
***Scapula Tilting***							
ROM	ET	4.81 (1.87)	4.93 (1.92)	4.24 (1.65)	4.75 (2.08)	————	1.000
	ST	4.95 (2.12)	4.67 (2.65)	4.90 (1.50)	4.82 (1.35)	————	1.000
PTA	ET	-14.06 (2.26)	-12.90 (0.20)	-13.93 (3.22)	-12.89 (1.56)	————	1.000
	ST	-12.66 (1.89)	-13.89 (3.85)	-13.56 (0.60)	-13.16 (0.81)	————	1.000
***Elbow Flexion-Extension***							
ROM	ET	27.71 (12.78)	27.04 (10.38)	26.67 (10.36)	27.65 (9.97)	————	1.000
	ST	27.84 (5.02)	28.50 (10.38)	27.21 (9.94)	26.84 (5.22)	————	1.000
PTA	ET	75.12 (14.16)	74.67 (8.62)	75.29 (7.72)	77.13 (10.17)	————	1.000
	ST	77.40 (12.20)	76.26 (15.32)	76.45 (10.79)	75.56 (15.14)	————	1.000
***Elbow Pronation-Supination***							
ROM	ET	12.98 (2.53)	12.21 (4.02)	12.19 (4.29)	12.30 (4.52)	————	1.000
	ST	12.48 (4.28)	11.83 (4.42)	12.37 (2.96)	12.02 (3.43)	————	1.000
PTA	ET	122.18 (10.74)	121.15 (12.12)	122.89 (14.64)	121.15 (11.84)	————	1.000
	ST	120.07 (17.94)	121.47 (10.89)	121.66 (15.27)	122.11 (12.87)	————	1.000
***Trunk Flexion-Extension***							
ROM	ET	3.36 (0.45)	3.44 (0.47)	3.32 (0.36)	3.15 (1.09)	————	1.000
	ST	3.25 (0.35)	3.34 (0.50)	3.31 (0.65)	3.20 (0.49)	————	1.000
PTA	ET	6.24 (1.19)	3.86 (0.86)*	6.32 (1.69)	6.17 (1.92)	2.58 (1.09 to 4.08)	0.001
	ST	6.27 (1.24)	3.08 (1.14)*	6.23 (1.04)	6.13 (1.15)	3.19 (2.03 to 4.35)	<0.001
***Trunk Lateral Flexion***							
ROM	ET	1.80 (1.19)	1.83 (0.87)	1.79 (0.50)	1.81 (0.79)	————	1.000
	ST	1.82 (0.98)	1.90 (1.17)	1.80 (1.16)	1.79 (0.89)	————	1.000
PTA	ET	1.55 (1.08)	1.59 (0.88)	1.56 (1.11)	1.74 (1.28)	————	1.000
	ST	1.62 (1.13)	1.56 (0.87)	1.73 (1.36)	1.56 (0.97)	————	1.000
***Trunk Axial Rotation***							
ROM	ET	4.37 (0.27)	3.76 (0.55)	4.20 (1.88)	4.52 (1.62)	————	1.000
	ST	3.47 (0.88)	4.56 (2.20)	4.15 (1.48)	4.22 (1.94)	————	0.742
PTA	ET	0.85 (1.62)	0.91 (1.98)	0.78 (2.21)	0.87 (2.48)	————	1.000
	ST	0.81 (2.94)	0.79 (2.97)	0.82 (2.07)	0.77 (3.33)	————	1.000

Data expressed as mean and standard deviation. ET: Elastic tape first. ST: Sham Tape first. Start: Starting angle. PTA: Point of Task Achievement. ROM: Range of Motion. CI: confidence interval (just for variable with significant interaction effect). p-value of comparison between pre and post elastic tape intervention for each group (ET and ST). ————: when p-value of interaction, group and time evaluation effects were not significant. NS: not significant (interaction, group and time evaluation effects).

*p<0.05 compared to pre-ET intervention.

Before elastic tape and sham tape intervention (baselines), groups are similar for starting angles of the shoulder elevation plane (p = 0.854; p = 0.679), scapula protraction-retraction (p = 0.961; p = 0.944) and trunk flexion-extension (p = 0.903; p = 0.936). Moreover, both groups did not present any difference between pre-elastic tape and pre-sham tape interventions for the starting angles of the elevation (ET: p = 1.00; ST: p = 1.00), scapula protraction-retraction (ET: p = 1.00; ST: p = 1.00) and trunk flexion-extension (ET: p = 1.00; ST: p = 1.00).

For the PTA angles, common interaction effects for all phases were observed (Tables 1–4), which consisted of more shoulder motion towards midline (for all phases: p<0.001, *η*^2^ = 0.32 [reaching], *η*^2^ = 0.41 [transporting to the mouth and returning]; *η*^2^ = 0.52 [transporting to the table]), less scapula protraction (for all phases: p = 0.002, *η*^2^ = 0.32 [reaching]; p = 0.001, *η*^2^ = 0.33 [transporting to the mouth]; p<0.001, *η*^2^ = 0.32 [transporting to the table]; p = 0.004, *η*^2^ = 0.26 [returning]), and less trunk flexion (for all phases: p<0.001, *η*^2^ = 0.48 [reaching], *η*^2^ = 0.42 [transporting to the mouth]; *η*^2^ = 0.44 [transporting to the table], *η*^2^ = 0.41 [returning]) when elastic tape intervention was made compared to sham intervention.

No differences were observed between the groups before elastic and sham tapes interventions for the PTA angles of the shoulder elevation plane, scapula protraction-retraction and trunk flexion-extension during the reaching phase (before elastic tape: p = 0.424; p = 0.961; p = 0.810/ before sham tape: p = 0.546; p = 0.809; p = 0.491), transporting the glass to the mouth (p = 0.973; p = 0.753; p = 0.887)/ p = 0.688; p = 0.947; p = 0.687, transporting the glass to the table (p = 0.897; p = 0.719; p = 0.345/ p = 0.631; p = 0.820; p = 0.413) and returning to the initial position (p = 0.782; p = 0.981; p = 0.984/ p = 0.952; p = 0.913; p = 0.955). Moreover, regarding time assessment, both groups (ET and ST) did not present any differences before the pre elastic and sham tape interventions for PTA angles of the shoulder elevation plane, scapula protraction-retraction and trunk flexion-extension during the reaching phase (all p = 1.0), transporting the glass to the mouth (all p = 1.0), transporting the glass to the table (all p = 1.0) and returning to the initial position (all p = 1.0).

Other specific interaction effects for PTA angles (Tables 1–4) were observed per phase. At the end of reaching phase, the elastic tape increased the scapula lateral rotation (p<0.001, *η*^2^ = 0.40) and elbow extension (p<0.001, *η*^2^ = 0.46). At the end of transporting the glass to the mouth, patients who underwent the elastic tape intervention presented more shoulder elevation (p = 0.001, *η*^2^ = 0.35) and less scapula lateral rotation (p = 0.001, *η*^2^ = 0.43). In addition, a medium elastic tape effect was observed at the elbow, an indication for increased elbow extension at the end of transporting the glass to the table (p<0.001 *η*^2^ = 0.44).

In addition, groups (ET and ST) were not different at baselines (before elastic and sham tape interventions) for PTA angles of scapula protraction-retraction (p = 0.961; p = 0.944) and elbow flexion-extension (p = 0.902; p = 0.921) during the reaching phase; for PTA angles of shoulder elevation (p = 0.912; p = 0.786) and scapula rotation (p = 0.753; p = 0.947) during transporting the glass to the mouth; and for PTA angles of elbow flexion-extension (p = 0.929; p = 0.726) during transporting the glass to the table. Regarding the time assessment comparisons, both groups did not present any differences between the pre-elastic tape application and pre-sham tape application for PTA angles of scapula protraction-retraction (all p = 1.0) and elbow flexion-extension (all p = 1.0) during the reaching phase; for PTA angles of shoulder elevation (all p = 1.0) and scapula rotation (all p = 1.0) during transporting the glass to the mouth; and for PTA angles of elbow flexion-extension (all p = 1.0) during transporting the glass to the table.

### Kinematic waveforms

SPM analysis revealed common differences between pre- and post-taping intervention for the elevation plane (more towards midline), less scapula protraction and trunk flexion while reaching (≈0–46% and 66–100%; 0–32% and 49–100%; 0–64% and 79–100%, respectively, S1B Fig), transporting to the mouth (51–100%; 66–100%; 83–100%, respectively, S1C Fig), transporting to the table (36–100%; 37–100%; 53–100%, respectively, S1D Fig) and returning (69–100%; 87–100%; 90–100%, respectively, S1E Fig). Moreover, more shoulder elevation and scapula lateral rotation during reaching (25–65% and 81–100%, respectively) (S1B Fig), as well as more shoulder elevation and less scapula lateral rotation while transporting to the mouth (20–100% and 15–100%, respectively, S1C Fig) was found. Intervention effects were also observed for the elbow joint (more extension) during reaching (54–100%, S1B Fig) and transporting to the table (84–100%, S1D Fig).

## Discussion

Although elastic tape has been widely used by physiotherapists, its effect on UL movements has been studied only recently [13–15, 19, 20, 34, 35]. To the best of our knowledge, this is the first study that evaluated its effect on movement strategies during a functional task for post-stroke patients. The results of the present study revealed that elastic tape did not immediately influence the spatiotemporal parameters of the drinking task in chronic hemiparetic subjects with mild to moderate UL impairments. However, ET intervention changed the shoulder position (more towards midline) and reduced scapula protraction and trunk flexion at the beginning, throughout, and at the end of the task, with small and medium effects. Moreover, using elastic tape increased shoulder elevation during reaching (for half the phase) and transporting the glass to the mouth increased the elbow extension near the table without and with the glass, increased the scapula lateral rotation (upward rotation) at the end of the reaching phase and decreased the scapula lateral rotation throughout the movement of bringing the glass to the mouth. Moreover, no adverse effects were observed in this study. Overall, these results demonstrated that elastic taping could alter UL movement strategies, thereby decreasing movement deviations in chronic hemiparetic individuals, taking age-gender matched healthy individuals as a reference [6].

The results of the present study are in line with the study by van Herzeele et al., who observed changes in scapular motion (i.e. higher posterior tilting and upward rotation) during humeral elevations in the sagittal, frontal, and scapular plane in athletes with elastic tape which was applied from the coracoid process to the thoracic spine process over the upper trapezius muscle compared to the condition without intervention [36]. Camerota et al. verified the effects of elastic tape on UL performance during reaching tasks in children with Cerebral Palsy. After 2 weeks of treatment, they observed a decreased movement duration, and improved smoothness, straightness of motion and ROM of the shoulder and elbow [37]. For stroke survivors, previous studies reported improved UL motor function, measured by FMA-UL [13] and Manual Function Task [19], after three and 28 weeks of elastic tape treatment, respectively. Along the same lines, a large effect of elastic tape was observed resulting in improved shoulder joint position sense in chronic hemiparetics after 10 minutes of using it [11].

These previous studies, combined with neuroscience paradigms suggest that the change in UL movement strategies when using elastic tape occurs due to an increased sensitive input by tactile stimulation, which is processed and integrated by the central nervous system that transforms sensory information into planned movements and calculates the necessary programs for movements (feedforward and feedback control). This process is known as sensorimotor integration [11, 12, 37]. Moreover, this improvement in neuromotor control can provided a better activation of shoulder girdle stabilizing muscles [12]. All these mechanisms may favor the motor schemes (greater perception of the UL in space), performance and (re) learning of more physiological movements [11, 18, 37, 38]. Furthermore, these effects can be attributed to the elastic property of tape as these improvements in UL performance were not observed when the patients were treated with non-elastic tape. This effect can be attributed to placebo effects; however, this application was the same as that performed in previous studies that performed a placebo validation to verify the expected effects by patients for each type of tape (elastic and non-elastic) and did not observe differences between the tapes.

Finally, as there were no changes in the JPS between the two evaluations on the non-paretic (i.e. non-treated) side, it can be concluded that there was no learning effect.

However, although the elastic tape changed joint motions in a short period, this influence varied from small to medium effects, without effects on spatiotemporal variables. Thus, these results point to using the elastic tape as an adjuvant intervention, especially when associated with movement therapy. For example, generating movements increases the sensory stimulation by the tape and other factors (i.e. visual stimulus), which reinforce all the previously mentioned mechanisms. According to the literature, elastic taping applications together with proprioceptive exercise stimulate a greater number of cutaneous receptors, which can enhance stability and joint control [12]. Moreover, it is suggested that the tape should be applied at the beginning of the therapy session to generate immediate effects on neuromotor control, which could maximize the gains obtained by motor learning. Thus, taping could ‘prime’ the central nervous system for subsequent motor tasks [39]. In a clinical setting, long-term effects are attributed to taping when it is not removed, which can be an important benefit; however, future studies need to focus on this aspect.

While the overall results suggest the benefits of using elastic tape as an intervention associated with other therapies in chronic hemiparetic subjects with mild to moderate UL impairments, it should be noted that the findings are task-specific. The results were limited to immediate effects of elastic tape on motor performance during the drinking task, as well as to that elastic tape brand (Kinesio Taping).

Moreover, the sample size of chronic hemiparetic subjects was small, with mild or moderate UL sensorimotor impairment. Future studies should include larger sample sizes and should focus on the effect of long-term elastic tape on UL performance, during different functional tasks, as well as on its effect as an adjuvant therapy, and its effect on stroke survivors with various degrees of UL impairment.

Whilst it was not the primary aim of the present study to compare the data analysis methods, it is important to highlight that the SPM analysis demonstrated the effects of the tape along the entire movement cycle. This extra analysis revealed information that was not observed by means of the extracted scalar kinematic parameters. For example, increased shoulder elevation was observed between 25% and 65% of the reach phase time, whilst no changes in extracted kinematic parameters were found. This underlines the benefits of including this complementary analysis to verify the joint motion strategies used during a UL task.

## Conclusion

Elastic tape provided small to moderate immediate changes in angular parameters during functional task in chronic post-stroke patients 10 minutes after the application in a single therapy session. For example, shoulder more toward midline, trunk less flexed and less scapula protraction were observed after elastic tape intervention. However, elastic tape did not alter spatiotemporal parameters and ROM of trunk, scapulothoracic, humerothoracic and elbow motions during functional task. Thus, these results could point to the inclusion of elastic tape as an adjuvant therapy and a ‘prime’ of the brain for subsequent motor training.

## Supporting information

S1 Fig Summarized presentation of SPM results (one example). The first graph shows the mean kinematic value of the elevation plane during waveforms when reaching for a glass at pre (black line) and post (blue line) elastic tape intervention. The middle graph presents SPM{t} as a function of the reaching phase. The critical threshold (t* = 3.386) was exceeded between 0–16%, 19–46% and 66–00% of the reaching phase. The black bar below the graph represents the time during which the differences between the evaluation time occurred (p<0.05), which was indicated by the SPM{t} statistic.
(TIF)

S1 Table Demographic characteristics of patients. Age expressed as mean (standard deviation), time post-stroke as mean (minimum-maximum) and total score of FMA-UL as median (maximum-minimum).
(DOC)

S1 File CONSORT checklist. (DOC)

S2 File Study protocol. (DOC)

S3 File Demographic characteristics of each subject. Individual data for all participants.
(XLS)

S4 File Spatiotemporal data, ROM, starting angles, and PTA for all joints assessed while reaching for a glass for both groups (ET and ST) pre and post-interventions (elastic and sham tape). (XLS)

S5 File Spatiotemporal data, ROM, and PTA for all joints assessed while transporting the glass to the mouth for both groups (ET and ST) pre and post-interventions (elastic and sham tape). (XLS)

S6 File Spatiotemporal data, ROM, and PTA for all joints assessed while transporting the glass to the table for both groups (ET and ST) pre and post-interventions (elastic and sham tape). (XLS)

S7 File Spatiotemporal data, ROM, and PTA for all joints assessed while returning to the initial position for both groups (ET and ST) pre and post-interventions (elastic and sham tape). (XLS)
