# Supplementary figures and images for: Effects of elastic tape on kinematic parameters during a functional task in chronic hemiparetic subjects: A randomized sham-controlled crossover trial
Source: PLoS One. 2019 Jan 25;14(1):e0211332. doi: 10.1371/journal.pone.0211332 (PMC6347187; doi:10.1371/journal.pone.0211332)

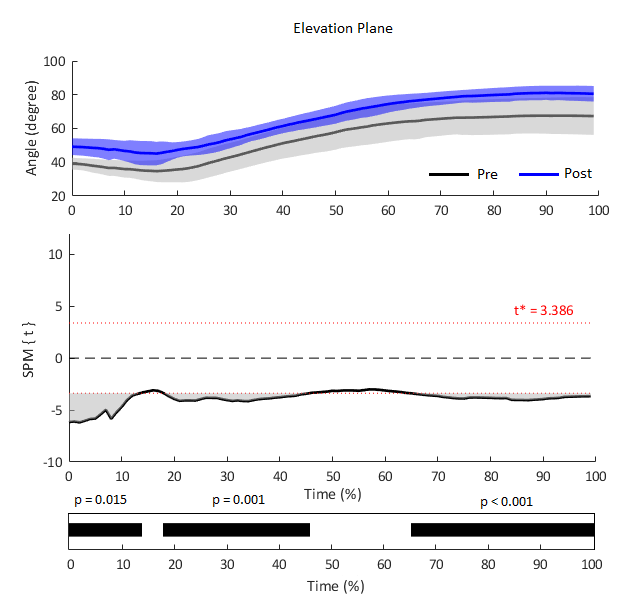

Supplement: S1 Fig — The first graph shows the mean kinematic value of the elevation plane during waveforms when reaching for a glass at pre (black line) and post (blue line) elastic tape intervention. The middle graph presents SPM{t} as a function of the reaching phase. The critical threshold (t* = 3.386) was exceeded between 0–16%, 19–46% and 66–00% of the reaching phase. The black bar below the graph represents the time during which the differences between the evaluation time occurred (p<0.05), which was indicated by the SPM{t} statistic. (TIF) [file pone.0211332.s001.tif]
