# Supplementary material for: Effects of elastic tape on kinematic parameters during a functional task in chronic hemiparetic subjects: A randomized sham-controlled crossover trial
Source: PLoS One. 2019 Jan 25;14(1):e0211332. doi: 10.1371/journal.pone.0211332 (PMC6347187; doi:10.1371/journal.pone.0211332)
Supplement: S1 Table — Age expressed as mean (standard deviation), time post-stroke as mean (minimum-maximum) and total score of FMA-UL as median (maximum-minimum). (DOC) [file pone.0211332.s002.doc]

**S1A Table – Demographic characteristics of patients.**

| **Demographic variables** | **Values** |
| --- | --- |
| Age (years) | 59.46 (8.88) |
| Gender (M/F) | 3/10 |
| Time post-stroke (months) | 75.23 (24-162) |
| Side of the injured hemisphere (R/L) | 7/6 |
| Dominant side before stroke (R/L) | 13/6 |
| Total score of FMA-UL | 49 (32-57) |

Ages expressed as mean (standard deviation), time post-stroke as mean (minimum-maximum) and total score of FMA-UL as median (maximum-minimum).

S1B Table - Spatiotemporal variables for all phases and both sequences (groups: ET and ST) pre and post-interventions (elastic and sham tape)

| Variables | Sequence | **Interventions** | | | | Statistic | | | | | | | |  |
| --- | --- | --- | --- | --- | --- | --- | --- | --- | --- | --- | --- | --- | --- | --- |
|  |  | Elastic Tape | | Sham Tape | | Interaction effect | | **Time effect** | | | Group effect | | |  |
|  |  | **Pre** | **Post** | **Pre** | **Post** | **F** | **p** | | **F** | **p** | | **F** | **p** | |
| ***Reaching for the glass*** | | | | | | | | | | | | | |  |
| PD (s) | ET | 1.52 (0.14) | 1.49 (0.27) | 1.39 (0.08) | 1.42 (0.21) | 0.18 | 0.91 | | 2.29 | 0.10 | | 1.19 | 0.10 | |
|  | ST | 1.53 (0.23) | 1.58 (0.37) | 1.70 (0.34) | 1.59 (0.36) |  |  |  |  |  |  |  |  |  |
| %PD | ET | 26.31 (1.76) | 26.31 (2.48) | 25.42 (1.85) | 26.94 (2.49) | 0.23 | 0.87 | | 2.81 | 0.05 | | 0.19 | 0.67 | |
|  | ST | 24.97 (1.99) | 26.07 (2.05) | 26.12 (1.43) | 26.18 (1.63) |  |  |  |  |  |  |  |  |  |
| %TPV | ET | 39.36 (4.36) | 36.99 (4.08) | 41.24 (5.32) | 37.68 (6.11) | 0.04 | 0.97 | | 2.89 | 0.07 | | 0.01 | 0.92 | |
|  | ST | 42.02 (4.03) | 37.51 (6.47) | 39.04 (4.11) | 37.35 (2.31) |  |  |  |  |  |  |  |  |  |
| PV (mm/s) | ET | 531.91 (29.49) | 520.75 (37.94) | 513.04 (41.17) | 562.73 (56.11) | 0.11 | 0.90 | | 2.71 | 0.06 | | 0.07 | 0.79 | |
|  | ST | 514.57 (70.18) | 553.84 (75.02) | 516.84 (81.17) | 515.20 (19.94) |  |  |  |  |  |  |  |  |  |
| TD | ET | 1.46 (0.06) | 1.42 (0.16) | 1.41 (0.15) | 1.42 (0.21) | 1.43 | 0.25 | | 2.23 | 0.05 | | 0.09 | 0.78 | |
|  | ST | 1.45 (0.16) | 1.38 (0.14) | 1.60 (0.27) | 1.39 (0.27) |  |  |  |  |  |  |  |  |  |
| ***Transporting the glass to the mouth*** | | | | | | | | | | | | | |  |
| PD (s) | ET | 1.52 (0.21) | 1.52 (0.11) | 1.53 (0.29) | 1.50 (0.22) | 0.07 | 1.00 | | 0.01 | 1.00 | | 0.07 | 0.94 | |
|  | ST | 1.50 (0.12) | 1.52 (0.18) | 1.52 (0.26) | 1.50 (0.10) |  |  |  |  |  |  |  |  |  |
| %PD | ET | 24.93 (1.42) | 24.76 (1.21) | 24.61 (1.71) | 24.90 (3.43) | 0.17 | 0.84 | | 0.58 | 0.56 | | 0.01 | 0.92 | |
|  | ST | 24.16 (0.92) | 24.90 (3.43) | 25.19 (0.71) | 24.79 (2.26) |  |  |  |  |  |  |  |  |  |
| %TPV | ET | 24.53 (1.39) | 25.92 (3.98) | 26.56 (3.32) | 24.51 (1.67) | 0.21 | 0.89 | | 0.93 | 0.44 | | 0.15 | 0.70 | |
|  | ST | 26.04 (2.64) | 24.75 (2.99) | 24.81 (2.43) | 24.63 (3.73) |  |  |  |  |  |  |  |  |  |
| PV (mm/s) | ET | 345.78 (79.92) | 343.91 (66.79) | 345.67 (76.46) | 344.06 (65.00) | 0.04 | 1.00 | | 0.02 | 1.00 | | 0.01 | 0.99 | |
|  | ST | 345.08 (71.04) | 345.17 (52.16) | 345.01 (83.27) | 345.62 (89.49) |  |  |  |  |  |  |  |  |  |
| TD | ET | 1.46 (0.06) | 1.42 (0.16) | 1.41 (0.15) | 1.42 (0.21) | 0.14 | 0.82 | | 0.07 | 0.89 | | 0.01 | 0.91 | |
|  | ST | 1.45 (0.18) | 1.38 (0.14) | 1.60 (0.26) | 1.39 (0.27) |  |  |  |  |  |  |  |  |  |
| ***Transporting the glass to the table*** | | | | | | | | | | | | | |  |
| PD (s) | ET | 1.76 (0.12) | 1.74 (0.14) | 1.76 (0.18) | 1.75 (0.42) | 0.01 | 0.98 | | 0.04 | 0.99 | | 0.02 | 0.96 | |
|  | ST | 1.75 (0.30) | 1.74 (0.36) | 1.74 (0.22) | 1.75 (0.11) |  |  |  |  |  |  |  |  |  |
| %PD | ET | 26.76 (2.39) | 27.43 (1.12) | 27.16 (1.69) | 26.88 (0.95) | 0.08 | 0.97 | | 0.13 | 0.94 | | 0.05 | 0.94 | |
|  | ST | 26.71 (3.69) | 27.09 (3.10) | 26.8 (2.41) | 27.20 (1.86) |  |  |  |  |  |  |  |  |  |
| %TPV | ET | 40.29 (10.01) | 40.52 (10.58) | 40.27 (9.02) | 40.12 (9.61) | 0.09 | 0.89 | | 0.02 | 0.96 | | 0.01 | 0.99 | |
|  | ST | 40.73 (9.67) | 40.79 (10.80) | 40.30 (11.85) | 39.80 (12.22) |  |  |  |  |  |  |  |  |  |
| PV (mm/s) | ET | 354.67 (89.31) | 354.79 (70.30) | 354.63 (80.74) | 355.75 (90.12) | 0.02 | 1.00 | | 0.02 | 1.00 | | 0.01 | 0.99 | |
|  | ST | 354.99 (64.72) | 354.97 (72.99) | 354.97 (82.95) | 354.00 (76.78) |  |  |  |  |  |  |  |  |  |
| TD | ET | 1.12 (0.04) | 1.11 (0.06) | 1.11 (0.04) | 1.11 (0.09) | 0.15 | 0.82 | | 0.03 | 0.95 | | 0.14 | 0.71 | |
|  | ST | 1.12 (0.05) | 1.12 (0.05) | 1.11 (0.04) | 1.11 (0.05) |  |  |  |  |  |  |  |  |  |
| ***Returning to initial position*** | | | | | | | | | | | | | |  |
| PD (s) | ET | 1.29 (0.24) | 1.30 (0.13) | 1.32 (0.24) | 1.31 (0.34) | 0.04 | 0.96 | | 0.01 | 0.99 | | 0.02 | 0.97 | |
|  | ST | 1.28 (0.24) | 1.31 (0.27) | 1.30 (0.28) | 1.31 (0.29) |  |  |  |  |  |  |  |  |  |
| %PD | ET | 21.42 (2.63) | 21.55 (2.26) | 21.83 (3.28) | 21.43 (2.90) | 0.05 | 0.98 | | 0.08 | 0.97 | | 0.06 | 0.81 | |
|  | ST | 21.98 (2.97) | 21.94 (2.95) | 21.31 (2.97) | 21.94 (2.95) |  |  |  |  |  |  |  |  |  |
| %TPV | ET | 51.78 (6.29) | 51.69 (4.55) | 51.64 (7.80) | 51.61 (8.12) | 0.02 | 1.00 | | 0.04 | 1.00 | | 0.07 | 0.94 | |
|  | ST | 51.50 (7.23) | 51.44 (6.85) | 51.63 (8.67) | 51.24 (7.34) |  |  |  |  |  |  |  |  |  |
| PV (mm/s) | ET | 518.27 (48.72) | 518.17 (57.80) | 519.04 (57.02) | 519.04 (58.77) | 0.01 | 1.00 | | 0.02 | 0.99 | | 0.01 | 0.98 | |
|  | ST | 519.46 (48.47) | 519.18 (43.73) | 519.04 (45.86) | 518.13 (32.92) |  |  |  |  |  |  |  |  |  |

Data expressed as mean and standard deviation. ET: Elastic tape first group. ST: Sham Tape first group. PD: phase duration. %PD: relative phase duration. PV: peak velocity. %TPV: time to peak velocity. TD: trajectory deviation.
