# Supplementary material for: Effects of elastic tape on kinematic parameters during a functional task in chronic hemiparetic subjects: A randomized sham-controlled crossover trial
Source: PLoS One. 2019 Jan 25;14(1):e0211332. doi: 10.1371/journal.pone.0211332 (PMC6347187; doi:10.1371/journal.pone.0211332)
Supplement: S2 File — (DOC) [file pone.0211332.s004.doc]

**Effects of elastic tape on the shoulder’s sensorimotor control and proprioception of chronic hemiparetic subjects (Protocol)**

**Abstract** Stroke is the second cause of death and the first of disabilities in the world. Although spontaneous motor recovery is observed, around 50 to 70% of the hemiparetic upper extremity presents alterations of upper extremity, limiting the performance of daily activities even after 2 to 4 years of stroke. Recently used in neurological rehabilitation, the Elastic Tape is able to facilitate the sensorimotor recovery. However, its safety and efficacy concerning the treatment of post-stroke individuals still require further investigation. Thus, the objective of this project is to evaluate the immediate effects of ET applied to the paretic shoulder on proprioception during movements of abduction and flexion. Twenty chronic hemiparetics (HC) and twenty healthy control subjects (C), matched by gender and age, will participate in this study. The groups will be subdivided into sham, S (HCS: n=10; CS: n=10) and ET (HCET: n=10; CET: n=10). Sensorimotor of HC and upper limb dominance will be assessed using the Fugl-Meyer Assessment and the Edinburgh Handness Inventory, respectively. ET will be placed on the deltoid muscle (anterior, middle and posterior portions). Evaluation before and after ET application will be carried out. Shoulder proprioception will be measured by absolute error during flexion and abduction movements. For statistical analyses, normality and homogeneity tests will be performed. If the variables present a normal and homogeneous distribution, a Two-way Anova Test will be used. Otherwise, a non-parametric statistic will be used (Kruskal-Wallis Test). A significance level of 0.05 will be considered for all statistical tests.

**Background**

Stroke is the second cause of death and the first of disabilities in the world . Although spontaneous motor recovery is observed, around 50 to 70% of the post-stroke subjects present alterations involving upper limbs, which are related to functional lack and disuse of upper limbs even after 2 to 4 years of stroke . The presence of residual deficits in the upper limb is the main contributor to limitations during daily living activities, and possibly restriction of participation of this population ([Sveen, 1999](#_ENREF_60); [Desrosiers *et al.*, 2003](#_ENREF_14); [Faria-Fortini *et al.*, 2011](#_ENREF_16)).

The main activities of daily living caused by such residual deficits of the upper limbs are those related to self-care and food, which both require reaching movements . Reaching movement requires highly coordinated actions of muscles, to promote temporal and sequential adjustments of joint movements, which are constantly regulated by sensory afferents . Thus, during reaching movements, the sensorimotor system receives afferent information from muscles, joints, skin surface and vision, which are meant to ensure, through feedback and feedforward control, the proper performance of the task according to planning. In addition, if necessary, possible paths errors and performance can be corrected .

However, hemiparetic subjects presented alterations in reaching movements, which consist of an increase in shoulder abduction and a decrease in the speed of movement, in elbow extension, and in interarticular coordination between the shoulder and elbow. These changes are associated with muscle activation deficits, observed by continuous activation of the upper trapezius and a co-contraction of the anterior posterior and middle deltoid, during a drinking task . In addition to these joint and muscle disorders that impair the performance of the reaching movement, 50% of post-stroke hemiparetic subjects present proprioceptive deficits in the upper limb . According to previous studies, chronic hemiparetic subjects present proprioceptive deficits in the paretic limb during the internal and external shoulder rotation , and bilateral deficits during shoulder abduction and flexion, which can damage the sensorimotor reaching performance .

Given these characteristics found in hemiparetic subjects, several interventions are used in clinical practice in order to minimize proprioception deficits and improve sensorimotor control during functional activities of the upper limb. One of the techniques widely used in clinical practice and supposed to optimize upper extremity function is elastic tape ([Oh, 2013](#_ENREF_28); Van Herzeele *et al.*, 2013). However, there is a lack of studies to assess the real effect of using elastic tape in the upper limbs of hemiparetic subjects. One of the most popular methods of using elastic tape is Kinesio Taping, developed in 1996 by Kenso Kase .

Various effects on the sensory system are attributed to this technique, however these mechanisms are not clear in the literature. Some hypothetical effects can explain the action of elastic tape, which are related to the close relationship between the somatosensory and motor functions in the cortex . Thus, it is possible that tactile stimulus generated by elastic tape activates the skin receptors, which transform this stimulus into an action potential that reaches the cortex contralateral primary somatosensory via thalamus. The sensorimotor cortex has a connection with multimodal association areas of the secondary somatosensory cortex, which integrates information from various sensory modalities, such as visual and proprioceptive information. These areas are linked to multimodal motor association areas, such as the posterior parietal area, limbic and prefrontal cortex, that transform sensory information into planned movements and calculate the necessary programs for these movements. In addition, these areas have a connection with the primary motor cortex and premotor areas, which send efferent information to the spinal cord and muscles. Thus, this motor information can be modulated through sensory information by taping .

However, the evidence of the effects of elastic tape in neurological patients is still limited. The effects of elastic tape mostly come associating elastic tape with toxin botulinum or stretching, which makes it difficult to interpret the isolated effects of elastic tape . Moreover, most studies did not use the elastic. These studies that used a non-elastic tape for mechanical correction observed a decrease in lower glenohumeral subluxation in hemiplegic individuals in acute and chronic phases, an increase in the shoulder range of motion, a reduction in pain and a functional improvement after using the elastic tape .

Nevertheless, methodological problems can be observed in studies using the elastic tape, such as the lack the control and/or sham group, and the association with other interventions. Thus, sham-controlled randomized studies in order to characterize neuromuscular responses from using elastic tape are still necessary. This study can help us to understand the use of elastic tape in rehabilitation programs with chronic hemiparetic subjects due to its extensive use in clinical practice, which is associated to its low cost and ability to maintain its effects even after the physical therapy session. In addition, the effects of ET should be expanded to functionality, i.e., considering neuromuscular changes, using ET may improve individuals´ performance in daily life activities, such as reaching.

**Hypotheses:** Elastic tape will improve shoulder proprioception during abduction and flexion with the paretic side.

**Primary objectives:** To assess the immediate effects of the elastic band (ET) used on the paretic shoulder on the joint position during flexion and abduction of chronic hemiparetic and healthy individuals.

**Proposed methodology:**

*Participants*

Twenty chronic hemiparetics (HC) and twenty control healthy subjects (C), matched by gender and age, will participate in this study. All participants must sign a consent form and informed consent to participate.

Hemiparetic individuals who had an ischemic stroke more than 6 months ago and who are not participating in any rehabilitation program or any experimental studies will be included in the study. Subjects who have had more than one stroke may be included as the stroke involves the same hemisphere. They should be aged between 40 and 70 years. They must present a level of less than 2 spasticity on the Modified Ashworth Scale (MAS) for abductors and shoulder flexors; the ability to perform reaching movements; proper trunk control confirmed by the individual's ability to remain in a sitting posture without trunk support and arms for 1 minute . The subjects will be included in the control group if they are healthy and present age and gender matched with the hemiparetic group. For the two groups, a minimum score in the Mini Mental State Examination will be considered , according to the volunteer's level of education . All individuals should have normal vision or corrected to normal.

The following exclusion criteria (hemiparetic and control) will be: diabetes mellitus, ulcers or skin lesions; elastic tape adverse reactions (redness and itching); serious cardiovascular or peripheral vascular disease (heart failure, arrhythmias, angina pectoris or myocardial infarction); other orthopedic or neurological diseases that impair data collection; cognitive or communication impairments; shoulder pain during the test; a history of muscle or joint injuries at the shoulder complex or cervical joints (fractures or surgery); body mass index (BMI) greater than 28 kg/m^2^; abnormal sensitivity, understanding of aphasia, apraxia, hemineglect and/or plegia that impair understanding or task execution. Furthermore, individuals with a passive range of motion of the shoulder lower than 90° flexion, 30° extension and adduction were excluded. For the healthy control, individuals with unstable shoulders were excluded either if a sulcus sign was found or an apprehension test was positive , as well as, subjects with a score < 8 in the Basal Physical Activity Questionnaire .

*Experimental Design*

On the first day, screening to select the sample and clinical assessments will be carried out. Clinical assessment will entail an interview which includes collecting personal data, a physical examination (anthropometric data) and investigating upper extremity sensorimotor impairment and manual preference by the Fugl-Meyer Assessment and the Edinburgh Handedness Inventory, respectively. After being evaluated, the subjects will be selected randomly using a sealed and opaque envelope to allocate the groups to receive the first Sham Tape (S) or first Elastic Tape (ET). Thus, the groups will be subdivided into sham, S (HCS: n=10; CS: n=10) and ET (HCET: n=10; CET: n=10). The control group will be divided because previous studies observed that elastic tape changes the muscle activation pattern and joint position sense . On the second and third days, the joint position sense (JPS) will be evaluated. On these days, the following sequence will be used: evaluation before intervention (ET or S), 10 minutes with intervention in a resting position, evaluation after intervention without removing intervention. A one-week interval between the first and second days will be respected. After the second day of assessment, a wash-out period of one month will be provided. On the third day, the participants will be evaluated by determining the JPS with an intervention contrary to the first intervention.

*Joint Position Test*

JPS will be carried out using a dynamometer. The following instructions will be given to the patient: (1) the dynamometer will move your arm to a specific position, (2) you will remain in this position for ten seconds. Concentrate on the position and where you arm is, (3) the dynamometer will return your arm to the starting position, (4) the dynamometer will move your arm again, and (5) press the button to stop the machine when you notice that your arm reaches the previous position . The stop button will be pressed with the non-paretic hand or dominant side. Initially, one familiarization trial will be carried out. During the test, participants will be blindfolded to rule out visual cues and no communication will be allowed . The dynamometer will move each subject’s upper extremity passively at a ﬁxed rate of 2.0° per second from the starting position (0° of abduction or flexion) to the reference positions (30° and 60° of abduction and then 30° and 60° of flexion). The absolute error (in degrees) will be calculated as the difference between the indicated and reference positions .

The test will be carried out three times for each limb (paretic or non-paretic/ dominant or non-dominant), movement (abduction or flexion) and angle (30° or 60°), before and after using the elastic or sham tape. The order of movements and angles will be in random order to prevent possible learning effects, however the assessments always begin with the paretic limb and non-dominant sides.

*Intervention*

Placing will be performed after initial assessments by a single physiotherapist certified in the Kinesio® Taping (KT) method. Blue Kinesio® Gold Tex PF tape (5 cm wide) for the elastic tape and a Cremer tape strip (10 m in width) for the sham will be used. Elastic and sham tape will be placed on the paretic shoulder in hemiparetic subjects and the dominant side of the healthy control group.

The acromioclavicular joint will be considered as the initial anchor for the Elastic Tape, and as the final one, the point immediately below the insertion of the deltoid muscle. The anchors will present 2cm for all the participants, and the active zone will be equivalent to the distance between two anchors. The first tape will be placed on the anterior portion of the deltoid with the shoulder at 30° passive extension. The second tape will be placed on the middle portion of the deltoid with the shoulder at 30° of passive horizontal adduction. To place the third tape on the posterior deltoid, the limb will be positioned at 90° of passive flexion of the shoulder (Figure 3). The elastic tape will be placed on tension previously described as “paper tension” and it is equivalent to 10-15% of the total elastic tape tension .

Sham tape will be placed without tension, i.e., the tape will be completely removed from the paper, then placed above the acromioclavicular joint in sagittal plane. It will be used in the anchor region where there is no effective participation in the chosen method, according to previous literature .

**Perceived effects and believability**

The placebo validation will involve assessing the perceived effects and credibility by asking three questions to the volunteers, done after assessing the first proprioception assessment. The questions are: "Do you expect the effects of the treatment you receive to: 1- improve perception of the limb in space, 2- improve using the limb, 3- improve the sensitivity of the limb ", with options of responding yes or no. Each response has a value of 0 to 1 and no to yes, reaching a maximum of 3 (maximum treatment effect) .

**Data Analysis Methodology:**

The results will be evaluated considering 2 aspects:

1. Characterization by initially assessing the hemiparetic and healthy control groups with inter-group comparison (paretic limb versus control; non-paretic limb versus control). Variables that will be considered: absolute error of joint position sense test during shoulder flexion and abduction.
2. Comparison between pre and post ET or S application for both groups and inter-groups. Variables that will be considered: absolute error of joint position sense test during shoulder flexion and abduction.

**Changes from in trial study protocol**

1. **Experimental design and participants:** the control group was removed from the study and the study design changed to crossover. This type of design avoids bias in the results (effects of intervention) regarding the baseline differences between the groups.
2. **Sham intervention:** the sham tape changed to non-elastic tape (Cremer tape strip - 5 cm wide) and was placed in the same way as the elastic tape. This change was based on possible effects attributed to the elastic tape: an increase in afferent input by skin stimulus due to its elastic property.

Moreover, it is worth mentioning that the study submitted to Plos One is part of a larger project.

**References**

Baecke, J. A., Burema, J., & Frijters, J. E. (1982). A short questionnaire for the measurement of habitual physical activity in epidemiological studies. *Am J Clin Nutr, 36*(5), 936-942.

Booth, F. W., & Lees, S. J. (2006). Physically active subjects should be the control group. *Med Sci Sports Exerc, 38*(3), 405-406. doi: 10.1249/01.mss.0000205117.11882.65

Callaghan, MJ, McKie, S, Richardson, P, & Oldham, JA. (2012). Effects of patellar taping on brain activity during knee joint proprioception tests using functional magnetic resonance imaging. *Phys Ther, 92*(6), 821-830. doi: 10.2522/ptj.20110209

Carda, S., Invernizzi, M., Baricich, A., & Cisari, C. (2011). Casting, taping or stretching after botulinum toxin type A for spastic equinus foot: a single-blind randomized trial on adult stroke patients. *Clin Rehabil, 25*(12), 1119-1127. doi: 10.1177/0269215511405080

Carr, Janet H. (1998). *Neurological Rehabilitation, 2/e*: Elsevier India.

Chae, J., Yang, G., Park, B. K., & Labatia, I. (2002). Delay in initiation and termination of muscle contraction, motor impairment, and physical disability in upper limb hemiparesis. *Muscle Nerve, 25*(4), 568-575.

Dukelow, S. P., Herter, T. M., Moore, K. D., Demers, M. J., Glasgow, J. I., Bagg, S. D., . . . Scott, S. H. (2010). Quantitative assessment of limb position sense following stroke. *Neurorehabil Neural Repair, 24*(2), 178-187. doi: 10.1177/1545968309345267

Feigin, V. L., Forouzanfar, M. H., Krishnamurthi, R., Mensah, G. A., Connor, M., Bennett, D. A., . . . Murray, C. (2014). Global and regional burden of stroke during 1990-2010: findings from the Global Burden of Disease Study 2010. *Lancet, 383*(9913), 245-254.

Folstein, M. F., Folstein, S. E., & McHugh, P. R. (1975). "Mini-mental state". A practical method for grading the cognitive state of patients for the clinician. *J Psychiatr Res, 12*(3), 189-198.

Freitas, S. M., Gera, G., & Scholz, J. P. (2011). Timing variability of reach trajectories in left versus right hemisphere stroke. *Brain Res, 1419*, 19-33. doi: 10.1016/j.brainres.2011.08.039

Gomez-Soriano, J, Abian-Vicen, J, Aparicio-Garcia, C, Ruiz-Lazaro, P, Simon-Martinez, C, Bravo-Esteban, E, & Fernandez-Rodriguez, JM. (2014). The effects of Kinesio taping on muscle tone in healthy subjects: a double-blind, placebo-controlled crossover trial. *Man Ther, 19*(2), 131-136.

Gomez-Soriano, J., Abian-Vicen, J., Aparicio-Garcia, C., Ruiz-Lazaro, P., Simon-Martinez, C., Bravo-Esteban, E., & Fernandez-Rodriguez, J. M. (2014). The effects of Kinesio taping on muscle tone in healthy subjects: a double-blind, placebo-controlled crossover trial. *Man Ther, 19*(2), 131-136.

Griffin, A., & Bernhardt, J. (2006). Strapping the hemiplegic shoulder prevents development of pain during rehabilitation: a randomized controlled trial. *Clin Rehabil, 20*(4), 287-295.

Hayner, K. A. (2012). Effectiveness of the California Tri-Pull Taping method for shoulder subluxation poststroke: a single-subject ABA design. *Am J Occup Ther, 66*(6), 727-736. doi: 10.5014/ajot.2012.004663

Hunter, SM, & Crome, P. (2002). Hand function and stroke. *Reviews in Clinical Gerontology, 12*(01), 68-81.

Jaraczewska, E., & Long, C. (2006). Kinesio taping in stroke: improving functional use of the upper extremity in hemiplegia. *Top Stroke Rehabil, 13*(3), 31-42. doi: 10.1310/33ka-xye3-qwjb-wgt6

Kandel, Eric R, Schwartz, James H, & Jessell, Thomas M. (2000). *Principles of neural science* (Vol. 4): McGraw-Hill New York.

Karadag-Saygi, E., Cubukcu-Aydoseli, K., Kablan, N., & Ofluoglu, D. (2010). The role of kinesiotaping combined with botulinum toxin to reduce plantar flexors spasticity after stroke. *Top Stroke Rehabil, 17*(4), 318-322. doi: 10.1310/tsr1704-318

Kase, K., Wallis, J., & Kase, T. (2003). Clinical therapeutic applications of Kinesio Taping Method.

Kisiel-Sajewicz, K., Fang, Y., Hrovat, K., Yue, G. H., Siemionow, V., Sun, C. K., . . . Daly, J. J. (2011). Weakening of synergist muscle coupling during reaching movement in stroke patients. *Neurorehabil Neural Repair, 25*(4), 359-368. doi: 10.1177/1545968310388665

Lin, J. J., Hung, C. J., & Yang, P. L. (2011). The effects of scapular taping on electromyographic muscle activity and proprioception feedback in healthy shoulders. *J Orthop Res, 29*(1), 53-57. doi: 10.1002/jor.21146

Lodha, N., Naik, S. K., Coombes, S. A., & Cauraugh, J. H. (2010). Force control and degree of motor impairments in chronic stroke. *Clin Neurophysiol, 121*(11), 1952-1961. doi: 10.1016/j.clinph.2010.04.005

Massie, C. L., Malcolm, M. P., Greene, D. P., & Browning, R. C. (2012). Kinematic motion analysis and muscle activation patterns of continuous reaching in survivors of stroke. *J Mot Behav, 44*(3), 213-222. doi: 10.1080/00222895.2012.681321

Messier, S., Bourbonnais, D., Desrosiers, J., & Roy, Y. (2006). Kinematic analysis of upper limbs and trunk movement during bilateral movement after stroke. *Arch Phys Med Rehabil, 87*(11), 1463-1470. doi: 10.1016/j.apmr.2006.07.273

Michener, L. A., Kardouni, J. R., Lopes Albers, A. D., & Ely, J. M. (2013). Development of a sham comparator for thoracic spinal manipulative therapy for use with shoulder disorders. *Man Ther, 18*(1), 60-64. doi: 10.1016/j.math.2012.07.003

Michener, L. A., Kardouni, J. R., Sousa, C. O., & Ely, J. M. (2015). Validation of a sham comparator for thoracic spinal manipulation in patients with shoulder pain. *Man Ther, 20*(1), 171-175. doi: 10.1016/j.math.2014.08.008

Murphy, Margit Alt, Willén, Carin, & Sunnerhagen, Katharina S. (2011). Kinematic variables quantifying upper-extremity performance after stroke during reaching and drinking from a glass. *Neurorehabilitation and neural repair, 25*(1), 71-80.

Murray, C. J., Vos, T., Lozano, R., Naghavi, M., Flaxman, A. D., Michaud, C., . . . Memish, Z. A. (2012). Disability-adjusted life years (DALYs) for 291 diseases and injuries in 21 regions, 1990-2010: a systematic analysis for the Global Burden of Disease Study 2010. *Lancet, 380*(9859), 2197-2223. doi: 10.1016/s0140-6736(12)61689-4

Niessen, M. H., Veeger, D. H., Koppe, P. A., Konijnenbelt, M. H., van Dieen, J., & Janssen, T. W. (2008). Proprioception of the shoulder after stroke. *Arch Phys Med Rehabil, 89*(2), 333-338. doi: 10.1016/j.apmr.2007.08.157

Niessen, M. H., Veeger, D. H., Koppe, P. A., Konijnenbelt, M. H., van Dieën, J., & Janssen, T. W. (2008). Proprioception of the shoulder after stroke. *Archives of physical medicine and rehabilitation, 89*(2), 333-338.

Pandian, J. D., Kaur, P., Arora, R., Vishwambaran, D. K., Toor, G., Mathangi, S., . . . Arima, H. (2013). Shoulder taping reduces injury and pain in stroke patients: randomized controlled trial. *Neurology, 80*(6), 528-532. doi: 10.1212/WNL.0b013e318281550e

Rueda, F. M., Montero, F. M. R., Torres, M. P. H., Diego, I. M. A., Sanchez, A. M. , & Page, J. C. M. (2012). [Movement analysis of upper extremity hemiparesis in patients with cerebrovascular disease: a pilot study]. *Neurologia, 27*(6), 343-347. doi: 10.1016/j.nrl.2011.12.012

Santos, G.L., Salazar, L.F.G., Lazarin, A.C., & Russo, T.L. (2015). Joint position sense is bilaterally reduced for shoulder abduction and flexion in chronic hemiparetic individuals. *Topics in Stroke Rehabilitation, 22*(4), 271-280. doi: 10.1179/1074935714Z.0000000014

Schaechter, J. D., Kraft, E., Hilliard, T. S., Dijkhuizen, R. M., Benner, T., Finklestein, S. P., . . . Cramer, S. C. (2002). Motor recovery and cortical reorganization after constraint-induced movement therapy in stroke patients: a preliminary study. *Neurorehabil Neural Repair, 16*(4), 326-338.

Thorp, A. A., Owen, N., Neuhaus, M., & Dunstan, D. W. (2011). Sedentary behaviors and subsequent health outcomes in adults a systematic review of longitudinal studies, 1996-2011. *Am J Prev Med, 41*(2), 207-215. doi: 10.1016/j.amepre.2011.05.004

van Vliet, P. M., & Sheridan, M. R. (2007). Coordination between reaching and grasping in patients with hemiparesis and healthy subjects. *Arch Phys Med Rehabil, 88*(10), 1325-1331. doi: 10.1016/j.apmr.2007.06.769

Wilk, K. E., Andrews, J. R., & Arrigo, C. A. (1997). The physical examination of the glenohumeral joint: emphasis on the stabilizing structures. *J Orthop Sports Phys Ther, 25*(6), 380-389. doi: 10.2519/jospt.1997.25.6.380

Williams, G. R. (2001). Incidence and characteristics of total stroke in the United States. *BMC Neurol, 1*, 2.

Yalcin, Elif, Akyuz, Mufit, Onder, Burcu, Kurtaran, Aydan, & Buyukvural, Sidika. (2012). Position Sense of the Hemiparetic and Non-Hemiparetic Ankle after Stroke: Is the Non-Hemiparetic Ankle also Affected? *European neurology, 68*(5), 294-299.

Zackowski, K. M., Dromerick, A. W., Sahrmann, S. A., Thach, W. T., & Bastian, A. J. (2004). How do strength, sensation, spasticity and joint individuation relate to the reaching deficits of people with chronic hemiparesis? *Brain, 127*(Pt 5), 1035-1046. doi: 10.1093/brain/awh116
